# Eye Movement Desensitisation and Reprocessing (EMDR) therapy for prolonged grief: theory, research, and practice

**DOI:** 10.3389/fpsyt.2024.1357390

**Published:** 2024-04-15

**Authors:** Liam Spicer

**Affiliations:** ^1^ School of Psychology, Faculty of Health Sciences, Curtin University, Perth, WA, Australia; ^2^ School of Psychological Sciences, College of Health and Medicine, University of Tasmania, Launceston, TAS, Australia

**Keywords:** grief, EMDR, prolonged grief, trauma, therapy, mental health

## Abstract

Prolonged Grief Disorder occurs within 7-10% of the bereaved population and is a more complicated and persistent form of grief which has been associated with suicidality, mental health disorders, sleep disturbance, poor health behaviors, and work and social impairment. EMDR is a fitting treatment option for those with Prolonged Grief, focusing on processing past memories, blocks, current triggers, future fears, and preparing the person for living life beyond the loss in line with the Adaptive Information Processing Model and grief frameworks. This paper discusses the theory, research regarding the application of EMDR with prolonged grief, and gives insight and guidance to clinicians working in this area including a case example.

## Introduction

Despite the universality of death and bereavement, losing a loved one is often a major distressing event in an individual’s life. Although the pain may remain in some form, often over time the loss of someone is accommodated into an individual’s life, and they can live a life of purpose and meaning beyond the loss. Research has demonstrated however that 7-10% of the bereaved population develop a more complicated and prolonged grief response (1). Although various terms have been used for this grief response, there is general consensus now among experts in the field that Prolonged Grief Disorder (PGD) is the correct classification for this response, now being included in both the International Classification of Diseases, 11^th^ Edition (ICD-11) and Diagnostic and Statistical Manual of Mental Disorders, Fifth Edition, Text Revision (DSM-5-TR). Prolonged Grief includes various symptoms and challenges such as difficulty accepting the death, a sense of meaninglessness, yearning for the deceased and associated emotional pain and difficulty engaging in new activities, with a diagnosis only being warranted after a minimum 6–12-month time frame. Although there is no direct research on the duration of PGD, it is commonly understood this disorder can last years and decades without significant improvement in symptoms (2). PGD has been associated with increased rates of suicidality, mental disorder and sleep disturbance, poor health behaviors, and work and social impairment (3–8). Due to the functional impact, increasing rates, and challenges associated with having a prolonged grief response, identifying individuals at risk, and those who need evidenced based treatments is of great importance. This notion is further supported by the findings by Lichtenthal et al. (9) demonstrating those with prolonged grief are less likely to receive support, despite being the ones in greatest need for intervention. Furthermore, Johnson et al. (10) demonstrated that more than 90% of individuals experiencing prolonged grief symptoms are relieved to understand their grief is a more complicated form, and report interest in receiving intervention for their prolonged grief symptoms. Having effective and widely available treatments for those with prolonged grief is further supported by Eisma, Boelen, and Lenferink (11) which have suggested an increase in the rates of prolonged grief since the COVID-19 pandemic due to a lack of social support, limited access to professional grief support, and the individual not being able to engage in traditional grief rituals to assist them in accommodating and adjusting to the loss (saying goodbye, viewing the body, and burial).

Up to this point, the research surrounding interventions for PGD has largely been centered around cognitive behavioral therapy (CBT) interventions. This preference for CBT focused approaches in addressing PGD might stem from previous clinical recommendations, which classified prolonged grief as a type of major depressive disorder (12). While numerous studies have reported significant outcomes from such interventions, a notable percentage of subjects do not experience meaningful therapeutic benefits (13). For instance, Bryant et al. (2) found that post-treatment, 37.9% of participants undergoing CBT continued to meet the criteria for PGD. In more recent years, other more specific interventions have been developed for the treatment of Prolonged Grief Disorder, notably Complicated Grief Treatment (CGT; 14). Complicated Grief Treatment is typically provided in a structured weekly format, generally consisting of 16 sessions (15). CGT is divided into 4 major phases: (1) getting started which involves areas such as getting a grief history, providing psychoeducation, building up social support and various grief related behavioral tasks, (2) core revisiting sequence which involves imaginal exposure based exercises to connect with the grief, (3) midcourse review which involves an evaluation of treatment progress and (4) closing sequence which includes preparation for closure and completing any other necessary supports and interventions (16). Although CGT has been demonstrated to be more effective than are proven efficacious treatments for depression, including citalopram (15) and interpersonal therapy (14, 17), there is still an absence of large, repeated randomized controlled trials demonstrating consistent results. Furthermore, in some research response rates in CGT were only 51% (14), and of practical relevance to clinicians, many individuals that may be working with Prolonged Grief are firstly not aware of CGT being an intervention, and secondly would have to become additionally trained and supervised in this approach. It is also important to note that similar with many other clinical presentations such as anxiety and depression, having various different treatment approaches available is of benefit as some clients may respond better to certain treatments provided. Furthermore, often certain components of treatments may be combined and integrated based on a client’s needs and goals. As an example, therapists could integrate elements of the getting started phase of CGT to have a grief focused, expert designed structure to assist with their assessment and preparation work, and then choose to implement EMDR therapy to process distressing elements of the loss, rather than the imaginal exposure-based exercises included in phase 2 of CGT.

Prolonged Grief Disorder has been listed in both the DSM-5-TR and ICD-11 under the trauma and stressor related disorder categories, and many experts within the field including Shear et al. (18), view PGD as a trauma and stress response. Based on this, treatment approaches should view prolonged grief through a “trauma lens”, allowing the adaptive resolution of information which is impeding on the natural grieving process. This is further supported by the illustration that several elements of PGD show considerable overlap with Post Traumatic Stress Disorder (PTSD) symptoms including intrusive images of the deceased, engaging in avoidance behavior, feeling estranged from others, sleep disturbance, and difficulty concentrating. Differences however do exist such as PTSD more being associated with fear, whereas prolonged grief associated with yearning and sadness. Furthermore, the content of intrusive images in PTSD being related to the traumatic event, whereas in prolonged grief this can be related to the person or factors associated with the loss (19). It is clear however that based on EMDR being a first line and consistently effective treatment for PTSD (20)., PGD being viewed as a trauma and stress response, and the overlap of PTSD and PGD, that the use of EMDR in this population is warranted. This is also supported by theoretical accounts and building empirical research as discussed below.

## Theory and frameworks: integrating our understanding of grief and the adaptive information processing model

In understanding a clients prolonged grief response, the Adaptive Information Processing Model (21) can act as our underlying framework in understanding as to why a clients natural grieving process has become blocked, complicated, or prolonged in some way. The Adaptive Information Processing Model suggests that mental health challenges and pathology is related to blocked information processing at the time of a traumatic, distressing, or challenging experience, and the subsequent way this information is stored maladaptively within our brain and nervous system. Due to the nature of an event, past history and experiences, and environmental and contextual factors at the time of an experienced event, this may impact on our ability to make sense and process an event or series of events that have occurred. If so, this information frozen and stored in a raw and state specific form can continue to impact on the way we see ourselves, the world, and our interactions with others, and through our engagement with life can be activated on a conscious or unconscious level leading to activation of images, feelings, thoughts, emotions, and subsequently certain behaviors.

In applying the AIP Model in the case of prolonged grief, integrating this theoretical framework with well-known and supported grief frameworks can add greater context to a client’s grief experience, and importantly give insight as to how EMDR therapy should be utilized. One of the most common, and well supported grief frameworks is known as the Dual Process Model by Stroebe and Schut (22). This model suggests that to be able to adjust and accommodate the loss into our lives, there needs to be a balance and oscillation between loss-oriented tasks and restoration-oriented tasks. In relation to this model, loss-oriented tasks involve aspects of the grief experience such as doing the active grief work, thinking about, and connecting with the loved person, engaging with reflection around what life was like before the loss, and connecting with memories and experiences regarding the person and life you had with them. The ultimate aim and goal of dealing with this area is to emotionally process the loss, involving a whole range of feelings, which does not occur in stages, but happens flexibly at certain time points (23). Loss oriented aspects of the grief are often more predominant during the initial phases of a loss, and this model considers factors associated with this such as closeness of the relationship, attachment, and other factors that may impede on engagement within this domain such as avoidance. Restoration oriented activities and engagement are focused on what life is like now, and what it will look like beyond the loss, involving components such as figuring out your new life role and identity now that this person is not here, creating new behaviors and experiences, forging new relationships, and recreating a life with meaning and fulfilment, whilst still keeping a connection with the deceased in some way if desired. This important area also includes dealing with secondary loss, such as the loss of an emotional support person due to the bereavement, feelings of isolation, and additional life changes contributing to distress such as increased household responsibilities, or life roles (24).

If we integrate these models as a unified framework for understanding prolonged grief, we can utilize our knowledge of risk factors in EMDR therapy to gain greater understanding of the possible blocks, information, and contributing factors that may impact or impede on someone’s natural healing process. Regarding loss-oriented tasks, there are several factors that have been consistently demonstrated in the research regarding prolonged grief that may impact on engagement in this domain. These include nature of the relationship with the deceased, childhood trauma, prior experiences of loss and pre-death mental health, attachment style, and schemas, (25–27). As an example, if an individual has prior unprocessed childhood trauma, their capacity to engage in sitting with feelings and processing the loss may be impacted due to challenges with managing distress, therefore there may be a need within our treatment to process this in addition to the grief related memories. Similarly, those with an insecure or avoidant attachment may want to avoid anything to do with loss-oriented tasks, and those with a self-sacrifice schema may put others needs for grieving over their own. Similarly, regarding restoration-oriented activities, various factors identified in the research may impact or impede on someone’s ability to adjust and accommodate the loss. These include areas such as low levels of social support, poorer family functioning, and low levels of optimism (28), considerations we need to take into account throughout our EMDR assessment, preparation and processing phases. As demonstrated, from combining the AIP model, Dual Process Model and identified risk factors associated with prolonged grief, our case conceptualization and knowledge of targets for processing in EMDR can be strengthened.

## EMDR and grief: what the research says

The use of EMDR with bereaved individuals is not uncommon, with Luber (29) outlining a suggested protocol for grief which involved discussing important considerations for clinicians regarding target assessment, and various goals and components of consideration when using EMDR for grief including connecting with a balanced range of emotions, and to accommodate the loss into an individual’s life. Solomon and Rando (30, 31) provided important insights for clinicians through multiple papers highlighting the importance of implementing EMDR within existing grief frameworks, considering all three prongs of EMDR reprocessing (past, present, and future), the need to focus on early life experiences and attachment as necessary, and the illustration of these principles through various case examples. In addition, Solomon and Hensley (32) discuss the use of EMDR for grief in the context of COVID-19 and more recently Solomon and Meysner (33) cover the use of EMDR with traumatic grief in the Oxford Handbook of EMDR, providing suggestions and guidance for clinicians working with traumatic bereavement. Regarding clinical research, Hornsveld et al. (34) investigated the efficacy of eye movements in reducing the emotionality of memories relating to loss. Sixty participants were asked to recall a negative loss-related memory before and after one of three conditions—eye movement, relaxation music, or a control with recall-only. In comparison to the relaxation music and control group, the eye movement group showed significantly greater reductions in both the ability to focus on the loss related memory and emotionality of the memory. In research by Sprang (35), 50 participants self-selected either EMDR or guided morning (exposure-based grief intervention) for individuals experiencing traumatic grief. Both treatments resulted in significant reductions in outcome measures such as reexperiencing, nightmares, rumination, and intrusive symptoms. This is consistent with Ironson et al. (36) findings in a PTSD population, however, EMDR participants experienced near complete symptom reduction at a much faster rate (8 sessions) in comparison to the GM condition in 13 sessions, an important finding for time-limited clinical settings. Furthermore, those who engaged in EMDR reported significant increases in positive memories of their loved ones, a finding not demonstrated in the GM condition, demonstrating additional benefits to EMDR as a grief intervention.

In another study by Meysner, Cotter, & Lee (37) the effectiveness of EMDR was compared with an integrated cognitive behavioral therapy (CBT) intervention for grief. Nineteen participants (12 females and 7 males) who identified themselves as struggling with grief were randomly allocated to treatment conditions for a 7-week treatment intervention. The results demonstrated CBT and EMDR to be equally effective in reducing grief symptoms, trauma symptoms, and distress. In a study using the same participants by Cotter, Meysner, and Lee (38), qualitative feedback on the benefits of each treatment were provided by participants. Participant reports common to both therapies included increased behavioral engagement, a better and heathier relationship to their deceased loved one, an increase in positive emotions, and an improvement in confidence. Regarding reported differences between each condition, the CBT group described benefits such as learning various emotional regulation skills and feeling like they were now in a new chapter of their life. In the EMDR condition, it was reported a noticeable benefit was that previously disturbing memories were now more distant and clearer.

In 2018 van Denderen et al., (39) completed a randomized control trial on the use of an integrated EMDR and CBT intervention for homicidally bereaved Dutch adults. The research included 85 participants, and demonstrated an integrated CBT and EMDR intervention was effective in reducing both complicated grief symptoms and Post Traumatic Stress Disorder (PTSD) symptoms. In a further similar study completed by Lennefrink et al. (40) a combined Cognitive Therapy and EMDR intervention was used with individuals bereaved by a loss from the MH17 plane crash. In their multicenter randomized control trial, self-rated scores for persistent complex bereavement, depression, and PTSD were comparted between an immediate treatment and waitlist control in 39 Dutch adults. The immediate treatment group showed a significant decline in depression, however no significant between group differences were observed regarding PTSD and grief symptoms.

More recently, (41) presented a case series on the efficacy of short term EMDR with patients with persistent complex bereavement. Three patients who had all lost a first degree relative were included in the case series, with between 1-2 sessions of EMDR being used to process complicated grief targets. In all cases positive improvements were noted on both quantitative clinical instruments, and feedback including changes such as not feeling guilty about the loss, being able to engage in life more, decreased avoidance, and returning to normal routines. In 2020 Solomon and Hensley discussed the application of EMDR in relation to grief and mourning in times of COVID-19. Similar to previous discussed findings, the case presented in this paper demonstrated positive improvement in symptoms and being able to move forward from the loss.

## EMDR for grief treatment considerations

EMDR therapy is a transdiagnostic and integrative psychotherapy (42) and as highlighted has been applied effectively with individuals experiencing various forms of grief. Regarding the application of EMDR with Prolonged Grief, the standard protocol is applied with consideration of all three prongs, and other targets as highlighted that may impact on the individuals natural grieving process. Furthermore, there are important considerations and areas of focus to be aware of as a clinician. Firstly, as previously discussed, in the assessment phase of EMDR when a client has experienced grief, it is of vital importance to understand and assess for the risk factors mentioned to understand a client’s current likelihood of developing a more complicated and prolonged grief response and areas to process within treatment. Regarding our education, not only is it important to frame the clients’ current challenges in line with the AIP mode, incorporating grief frameworks such as the Dual Process Model is essential. In preparation for processing, standard EMDR resource development (43) and self-regulation techniques (44) as necessary can be employed. In addition, considering restoration-oriented activities as mentioned and the risk factors that may impact on this area, building up social support, and creating hope and optimism through resource development may be necessary components to enhance clinical efficacy. Previous clinical research has also demonstrated that a positive therapeutic alliance early on in therapy results in a greater reduction of prolonged grief symptoms (45). Considering this as a core tenet of our approach aligns with recent peer reviewed articles by Piedfort-Marin (46) and Hase and Brisch (47) which highlight the core role of the therapeutic relationship in EMDR.

Regarding target assessment, and subsequently desensitization and reprocessing, prior memories that are resulting in the client not being able to grieve or accommodate the loss are often a key focus point of treatment. These targets can often be centered around the themes of responsibility (e.g., I should have done more) or an individual’s ability to cope (I can’t manage this), although each individual may be unique regarding how they process loss related information and previous events underlying their symptoms. As previously discussed, in line with the AIP model, there may be a need to process other past traumatic events, other losses, or other sources of maladaptively stored information impacting or impeding on an individual’s natural grieving process. Furthermore, in working with grief, significant importance is placed on current triggers and the use of the Flash Forward Approach (48) to process catastrophic or significantly disturbing fears towards the future. Some examples of current triggers may be someone talking about the loved one, being reminded of them from a song or place, or other loss associated material. Processing fears the client may have towards the future (e.g., being alone forever, never enjoying a moment again in life) with the flashforward technique often provides further beneficial effects for clients, allowing them to feel more comfortable about life in the future. Future templates also assist in this way, providing further adaptive information for the person to feel they can cope, keep a connection with their loved one, and still have a sense of meaning in their life. As highlighted, the AIP model and the application of EMDR across all 8 phases can be a fitting and appropriate treatment framework and approach for working with clients whose grief has become complicated and blocked in some form.

## Case example

Mary is a 24-year-old who lost her mother in a workplace accident. Since the death Mary has lost interest and engagement with hobbies, connecting with friends, avoids any reminders of the loss including places and people, and feels like life has lost all meaning and purpose. Mary has not engaged in therapy or talked about the grief with anyone for over 3 years. On assessment at the start of therapy, Mary met the PGD criteria based on the Prolonged Grief-13 (PG-13), a diagnostic tool to assess against the criteria for diagnosis. In EMDR therapy utilizing the standard protocol approach, a detailed history was taken with curiosity and assessment of known risk factors for PGD as highlighted earlier, and an assessment of other traumatic experiences which may be impacting on Mary’s current grief experience. Concurrently with gaining information about Mary’s history, and current challenges regarding the grief, Mary focused on building self-regulation skills through EMDR based techniques such as Resource Development and Installation (43). In addition, boosting up social connection and engagement was a major early focus, and from the preparation phase of EMDR, overall a willingness to start to process components of the grief and past trauma became more evident. This was in addition to Mary starting to have a greater positive connection to her deceased mother, being able to share positive stories and describe things that she loved about her in therapy. EMDR target assessment phase revealed certain EMDR targets for processing including the moment she found out about the death, a previous argument a few days before the death with her mother, previous losses and trauma from childhood, current triggers such as driving past her Mums house or work, and also fears of the future around what life would look like, in addition to positive future template work around being able to cope and manage events such as Christmas, anniversaries, and her Mother’s birthday. Mary responded well to treatment after 12 sessions of EMDR, no longer satisfying the criteria for a diagnosis of PGD. Mary showed improvement around being able to talk about the death or being in certain places related to the loss, has increased her connection with her mother in a way that feels meaningful to her, has been able to connect socially and with hobbies again, and has regained a sense of purpose in her life.

## Conclusion

Overall, EMDR is a powerful treatment approach for those whose natural grieving process has become blocked in some way. The AIP model in combination with the Dual Process Model provides a useful framework to understand what has caused these blocks, and how we can support clients in moving forward in life after loss. Although the research in this area is building, there needs to be further research completed, especially considering individuals who have experienced trauma are more at risk of developing a prolonged and complicated grief response. The research so far has been limited in terms of not examining the use of EMDR specifically for those who meet the diagnostic criteria for Prolonged Grief Disorder. Most studies have also mentioned limitations regarding sample size and follow up. Furthermore, no trials completed so far have compared EMDR with Complicated Grief Treatment by Katherine Shear (49), which currently has the most empirical support for this population group. Further studies examining the comparison of EMDR and CGT independently and also combined would be of benefit to determine whether there is any additional benefit of combining both, in comparison to just using one intervention.

## Author contributions

LS: Writing – original draft, Writing – review & editing.

## References

[1] Lundorff M Holmgren H Zachariae R Farver-Vestergaard I O’Connor M. Prevalence of prolonged grief disorder in adult bereavement: A systematic review and meta-analysis. J Affect Disord. (2017) 212:138–49. doi: 10.1016/j.jad.2017.01.030 28167398

[2] Bryant RA Kenny L Joscelyne A Rawson N Maccallum F Cahill C. Treating prolonged grief disorder: A randomized clinical trial. JAMA Psychiatry. (2014) 71:1332–9. doi: 10.1001/jamapsychiatry.2014.1600 25338187

[3] Szanto K Prigerson H Houck P Ehrenpreis L Reynolds CF III. Suicidal ideation in elderly bereaved: the role of complicated grief. Suicide Life-Threatening Behav. (1997) 27:194–207. doi: 10.1111/j.1943-278X.1997.tb00291.x 9260302

[4] Germain A Caroff K Buysse DJ Shear MK. Sleep quality in complicated grief. J Traumatic Stress: Off Publ Int Soc Traumatic Stress Stud. (2005) 18:343–6. doi: 10.1002/jts.20035 16281231

[5] Hardison HG Neimeyer RA Lichstein KL. Insomnia and complicated grief symptoms in bereaved college students. Behav Sleep Med. (2005) 3:99–111. doi: 10.1207/s15402010bsm0302_4 15802260

[6] Mitchell AM Kim Y Prigerson HG Mortimer MK. Complicated grief and suicidal ideation in adult survivors of suicide. Suicide Life-Threatening Behav. (2005) 35:498–506. doi: 10.1521/suli.2005.35.5.498 16268767

[7] Simon NM Pollack MH Fischmann D Perlman CA Muriel AC Moore CW. Complicated grief and its correlates in patients with bipolar disorder. J Clin Psychiatry. (2005) 66:1105–10. 10.4088/jcp.v66n0903 16187766

[8] Silverman GK Jacobs SC Kasl SV Shear MK Maciejewski PK Noaghiul FS. Quality of life impairments associated with diagnostic criteria for traumatic grief. psychol Med (2000) 30(4):857–62. 10.1017/s0033291799002524 11037094

[9] Lichtenthal WG Nilsson M Kissane DW Breitbart W Kacel E Jones EC. Underutilization of mental health services among bereaved caregivers with prolonged grief disorder. Psychiatr Serv. (2011) 62:1225–9. doi: 10.1176/ps.62.10.pss6210_1225 PMC3694810 21969652

[10] Johnson JG First MB Block S Vanderwerker LC Zivin K Zhang B. Stigmatization and receptivity to mental health services among recently bereaved adults. Death Stud. (2009) 33:691–711. doi: 10.1080/07481180903070392 19697482 PMC2834798

[11] Eisma MC Boelen PA Lenferink LI. Prolonged grief disorder following the Coronavirus (COVID-19) pandemic. Psychiatry Res. (2020) 288:113031. doi: 10.1016/j.psychres.2020.113031 32360895 PMC7194880

[12] American Psychiatric Association. Diagnostic and statistical manual of mental disorders-IV-TR. 4th, Text Revision ed. Washington: DC: American Psychiatric Association (2000).

[13] Breen LJ Hall CW Bryant RA. A clinician’s quick guide of evidence-based approaches: Prolonged grief disorder. Clin Psychol. (2017) 21:153–4. doi: 10.1111/cp.12124

[14] Shear K Frank E Houck PR Reynolds CF 3rd. Treatment of complicated grief: A randomized controlled trial. J Am Med Assoc. (2005) 293:2601–8. 10.1001/jama.293.21.2601 PMC5953417 15928281

[15] Shear MK Reynolds CF Simon NM Zisook S Wang Y Mauro C. Optimizing treatment of complicated grief: A randomized clinical trial. JAMA Psychiatry. (2016) 73:685–94. 10.1001/jamapsychiatry.2016.0892 PMC5735848 27276373

[16] Shear MK Gribbin Bloom C. Complicated grief treatment: An evidence-based approach to grief therapy. J Rational-Emotive Cognitive-Behavior Ther. (2017) 35:6–25.

[17] Shear MK Wang Y Skritskaya N Duan N Mauro C Ghesquiere A. Treatment of complicated grief in elderly persons: a randomized clinical trial. JAMA Psychiatry. (2014) 71:1287–95. 10.1001/jamapsychiatry.2014.1242 PMC5705174 25250737

[18] Shear K Monk T Houck P Melhem N Frank E Reynolds C. An attachment-based model of complicated grief including the role of avoidance. Eur Arch Psychiatry Clin Neurosci (2007) 257:453–61. 10.1007/s00406-007-0745-z PMC2806638 17629727

[19] Szuhany KL Malgaroli M Miron CD Simon NM. Prolonged grief disorder: Course, diagnosis, assessment, and treatment. Focus. (2021) 19:161–72. 10.1176/appi.focus.20200052 PMC8475918 34690579

[20] de Jongh A de Roos C El-Leithy S. State of the science: Eye movement desensitization and reprocessing (EMDR) therapy. J Traumatic Stress (2024). 10.1002/jts.23012 38282286

[21] Shapiro F. Eye movement desensitization and reprocessing (EMDR): Basic principles, protocols, and procedures. New York City, US: Guilford Press (2001).

[22] Stroebe MS Schut H. Meaning making in the dual process model of coping with bereavement. In Neimeyer RA (Ed.), Meaning reconstruction & the experience of loss (pp. 55–73). American Psychological Association. doi: 10.1037/10397-003

[23] Schut MSH. The dual process model of coping with bereavement: Rationale and description. Death Stud. (1999) 23:197–224. 10848151 10.1080/074811899201046

[24] Stroebe M Schut H. The dual process model of coping with bereavement: A decade on. OMEGA-journal Death Dying (2010) 61(4):273–89. 10.2190/OM.61.4.b 21058610

[25] He L Tang S Yu W Xu W Xie Q Wang J. The prevalence, comorbidity and risks of prolonged grief disorder among bereaved Chinese adults. Psychiatry Res (2014) 219(2):347–52. 10.1016/j.psychres.2014.05.022 24924526

[26] Schaal S Jacob N Dusingizemungu JP Elbert T. Rates and risks for prolonged grief disorder in a sample of orphaned and widowed genocide survivors. BMC Psychiatry. (2010) 10:55. doi: 10.1186/1471-244X-10-55 20604936 PMC2914709

[27] Thimm JC Holland JM. Early maladaptive schemas, meaning making, and complicated grief symptoms after bereavement. Int J Stress Manage. (2017) 24:347.

[28] Thomas K Hudson P Trauer T Remedios C Clarke D. Risk factors for developing prolonged grief during bereavement in family carers of cancer patients in palliative care: a longitudinal study. J Pain Symptom Manage. (2014) 47:531–41. doi: 10.1016/j.jpainsymman.2013.05.022 23969327

[29] Luber M. Protocol for excessive grief. J EMDR Pract Res. (2012) 6:129–35.

[30] Solomon RM Rando TA. Utilization of EMDR in the treatment of grief and mourning. J EMDR Pract Res. (2007) 1:109–17.

[31] Solomon RM Rando TA. Treatment of grief and mourning through EMDR: Conceptual considerations and clinical guidelines. Eur Rev Appl Psychol. (2012) 62:231–9.

[32] Solomon RM Hensley BJ. EMDR therapy treatment of grief and mourning in times of COVID-19. J EMDR Pract Res. (2020) 14(3):162–74. doi: 10.1891/EMDR-D-20-00031

[33] Solomon R Meysner L. EMDR Therapy and Traumatic Grief (Clinical Guidelines). (2023) In Farrell D. Schubert S Kiernan M. The Oxford Handbook of EMDR. Oxford University Press.

[34] Hornsveld HK Landwehr F Stein W Stomp MP Smeets MA van den Hout MA. Emotionality of loss-related memories is reduced after recall plus eye movements but not after recall plus music or recall only. J EMDR Pract Res. (2010) 4:106.

[35] Sprang G. The use of eye movement desensitization and reprocessing (EMDR) in the treatment of traumatic stress and complicated mourning: Psychological and behavioral outcomes. Res Soc Work Pract. (2001) 11:300–20.

[36] Ironson G Freund B Strauss JL Williams J. Comparison of two treatments for traumatic stress: A community-based study of EMDR and prolonged exposure. J Clin Psychol. (2002) 58:113–28. 10.1002/jclp.1132 11748600

[37] Meysner L Cotter P Lee CW. Evaluating the efficacy of EMDR with grieving individuals: A randomized control trial. J EMDR Pract Res. (2016) 10(1):1–11. doi: 10.1891/1933-3196.10.1.2

[38] Cotter P Lee CW. Evaluating the efficacy of EMDR with grieving individuals: A randomised control trial. J EMDR Pract Res. (2016) 10:3.

[39] van Denderen M de Keijser J Stewart R Boelen PA. Treating complicated grief and posttraumatic stress in homicidally bereaved individuals: A randomized controlled trial. Clin Psychol Psychother. (2018) 25:497–508. 10.1002/cpp.2183 29479767

[40] Lenferink LI de Keijser J Smid GE Boelen PA. Cognitive therapy and EMDR for reducing psychopathology in bereaved people after the MH17 plane crash: Findings from a randomized controlled trial. Traumatology. (2020) 26:427.

[41] Usta FD Yasar AB Abamor AE Caliskan M. A case series: Efficacy of short term EMDR on patients with persistent complex bereavement disorder (PCBD). Eur Psychiatry. (2020) 41:S360–1.

[42] Dominguez SK. Farrell D Schubert S Kiernan, editors. The Oxford Handbook of EMDR. Oxford University Press (2023).

[43] Leeds AM Shapiro F. EMDR and resource installation: Principles and procedures for enhancing current functioning and resolving traumatic experiences. Brief Ther strategies individuals couples. (2000), 469–534.

[44] van Genugten L Dusseldorp E Massey EK van Empelen P. Effective self-regulation change techniques to promote mental wellbeing among adolescents: a meta-analysis. Health Psychol Rev. (2017) 11:53–71. 27796160 10.1080/17437199.2016.1252934

[45] Glickman K Katherine Shear M Wall MM. Therapeutic alliance and outcome in complicated grief treatment. Int J Cogn Ther. (2018) 11:222–33.

[46] Piedfort-Marin O. Transference and countertransference in EMDR therapy. J EMDR Pract Res. (2018) 12:158–72.

[47] Hase M Brisch KH. The therapeutic relationship in EMDR therapy. Front Psychol. (2022) 13:835470. 35712194 10.3389/fpsyg.2022.835470 PMC9197431

[48] Logie R De Jongh A. The “Flashforward procedure”: Confronting the catastrophe. J EMDR Pract Res. (2014) 8:25–32.

[49] Katherine SM Mulhare E. Complicated grief. Psychiatr Ann (2008) 38 10.

